# Gene co-expression network analysis identifies the hub genes associated with immune functions for nocturnal hemodialysis in patients with end-stage renal disease

**DOI:** 10.1097/MD.0000000000012018

**Published:** 2018-09-14

**Authors:** Hongwei Dai, Jiao Zhou, Bo Zhu

**Affiliations:** Department of Blood Transfusion, Suizhou Hospital, Hubei University of Medicine, Hubei, China.

**Keywords:** end-stage renal stage, gene expression, nocturnal hemodialysis, transcriptional factors

## Abstract

End-stage renal disease (ESRD) is the final stage of chronic kidney disease in which the kidney is not sufficient to meet the needs of daily life. It is necessary to understand the role of genes expression involved in ESRD patient responses to nocturnal hemodialysis (NHD) and to improve the immunity responsiveness. The aim of this study was to investigate novel immune-associated genes that may play important roles in patients with ESRD.

The microarray expression profiles of peripheral blood in patients with ESRD before and after NHD were analyzed by network-based approaches, and then using Gene Ontology (GO) and Kyoto Encyclopedia of Genes and Genomes pathway analysis to explore the biological process and molecular functions of differentially expressed genes. Subsequently, a transcriptional regulatory network of the core genes and the connected transcriptional regulators was constructed. We found that NHD had a significant effect on neutrophil activation and immune response in patients with ESRD.

In addition, Our findings suggest that *MAPKAPK3*, *RHOA*, *ARRB2*, *FLOT1*, *MYH9*, *PRKCD*, *RHOG*, *PTPN6*, *MAPK3*, *CNPY3*, *PI3KCG*, and *PYGL* genes maybe potential targets regulated by core transcriptional factors, including ARNT, C/EBPalpha, CEBPA, CREB1, PSG1, DAND5, SP1, GATA1, MYC, EGR2, and EGR3.

## Abstract 2

Supplemental Digital Content is available in the text

## 1 Introduction

Chronic kidney disease is an age-related global public health problem that affects nearly 15% of the adult population worldwide.^[1]^ The number of older patients with end-stage renal disease (ESRD) increased rapidly. ESRD is an important but complex syndrome, a direct result contributes to significant morbidity and mortality in this population.^[2]^ The best choice for treatment of ESRD is renal transplantation, which can significantly improve survival and quality of life.^[3]^ Unfortunately, the supply of transplanted kidneys is less than demand. Therefore, dialysis is currently the only therapy for ESRD until the proper donor appears for optimal renal replacement.

Several preliminary studies have shown that nocturnal hemodialysis (NHD) has many benefits in ESRD patients compared with conventional hemodialysis (CHD). Such as improving the quality of life,^[4]^ lowering serum phosphate levels^[5]^ and improving erythropoietin,^[6]^ and have a positive effect on uremia-associated inflammation.^[7]^ However, the conclusions were inconsistent.^[8]^ In addition to that, immune function is a main factor affecting the quality of life of ESRD patients infected with invaded pathogens. Therefore, exploring the impact of NHD on the immune functions in patients with ESRD will facilitate the progression of the treatment.

Recently, gene expression assays have been performed using biochip (also known as DNA microarray or DNA chip) to investigate complex biological process and molecular function in cells activity. Scientists use biochip to measure the expression levels of a large number of genes simultaneously or to genotype multiple regions of a genome. Weighted gene co-expression network analysis (WGCNA), also known as weighted correlation network analysis, is widely used in data mining methods, especially for studying biological networks based on pairwise correlations between variables. Although it can be applied to most high-dimensional datasets, it has been most widely used in genomic applications. It allows modules (clusters), intramodular hubs, and network nodes to be defined in the module membership to study the relationships between co-expression modules and compare the network topology (differential network analysis) of different networks.

In this study, by using previously published gene microarray expression data, we first identified the gene transcriptional modules for NHD and CHD in patients with ESRD. And this work has powerful and identical evidence supporting the difference between these 2 hemodialysis methods.

## 2 Materials and methods

### 2.1 Microarray data processing

Microarray dataset was collected from a previous study of peripheral blood samples obtained from Gene Expression Omnibus (GEO, http://www.ncbi.nlm.nih.gov/geo/) and GEO Series Accession NO. GSE11227. Samples were obtained from the tissue library of human peripheral blood samples from 16 patients with ESRD in the laboratory of Oncology Research, Toronto General Hospital, Toronto, Canada. The 16 samples were analyzed using a chip-based platform GPL570 (HG-U133_Plus_2) Affymetrix Human Genome U133 Plus 2.0 Array.^[9]^ Microarray data analysis was performed using R software and Bioconductor 3.5 (http://www.bioconductor.org/). All expression data were quantile normalized and log2 transformed by Robust Multiarray Averaging. In addition, this study follows the dissemination and application policy requirements of GEO public data and has been approved by the Ethics Committee of the Institute of Biomedicine Research of the Hubei University of Medicine.

### 2.2 Differentially expressed genes analysis

Differential expression genes analyses were carried out by DESseq2 and Bioconductor packages based on their normalized signal intensity in “NHD” and “CHD” profiles. To reduce the heterogeneity between different clinical samples, we obtained class 3 level datasets which was normalized to the same intensity values by quantile normalization and log2 transformation. Finally, we selected differentially expressed mRNAs with criterion of *P* value <.05 and FDR <0.05.

### 2.3 Weighted gene co-expression network analysis

WGCNA can be used to summarize the obtained modules by using the concept of eigengene and to further screen the appropriate gene targets by calculating module membership metric (also known as eigengene-based connectivity).^[10]^ In the data processing, the whole genome gene expression data were preliminarily filtered, and then the consistency of the gene expression spectrum was determined by Pearson correlation method. And the data were transformed to an adjacency matrix by using the soft threshold power beta, which propose covariant similarity. The power of beta = 10 was chosen based on the scale-free topology criterion. Finally, the topological overlap matrix (TOM) was calculated and the hierarchical clustering was used to generate dendrogram from a similarity TOM. By using dynamic tree cutting, different number of clusters (modules) were obtained from the tree.

We then analyzed the importance of the genes by *t*-test to determine whether the modules were associated with NHD. The module eigengene (ME) refers to the first principal component gene of module expression matrix. It is considered to be the most representative of the module genes, which has important biological significance.

### 2.4 Enrichment analysis of biological process

Gene ontology (GO) is a major bioinformatics program that is used to unify the expression of genes and gene product attributes for all species.^[11]^ More specifically, the project aims to maintain and develop its controlled vocabulary of gene and gene product attributes; annotate genes and gene products, and assimilate and disseminate annotation data; and provide tools to facilitate access to all aspects of the data provided by the project, for example, via enrichment analysis.

GO classification was used to explain the main function of differentially expressed genes (DEGs) according to the GO database,^[12]^ which is the crucial functional classification of NCBI.^[13]^ Fisher exact test was used to calculate the significance level of each term to screen out the important terms for DEGs enrichment. The statistical significance (*P* < .01) of GO terminology in GO analysis was chosen, and the GO enriched map was constructed using the upregulation and downregulation to summarize the impact of experimental function of ESRD. Through the establishment of functional relationship network, it was possible to summarize the impact of the experimental function group and the important characteristics of internal affiliation. Finally, we selected the differently expressed mRNAs with the *P* value < .05 criterion.^[14]^

### 2.5 Transcriptional regulatory network of hub genes

The hub genes are associated with the highest degree of a series of genes in the module, which are described as the most closely related to more biological significance. To identify the hub genes in each module genes, we calculated their in-module connectivity from the signed TOM based on an adjacency matrix. Then we extracted the most powerful connection in the module with a proper threshold. University of California, Santa Cruz (UCSC) (http://genome.ucsc.edu/) is an open-access database for predicting all transcription factors (TFs) to potential target relationships based on reference gene files which also came from UCSC. Subsequently, we constructed a network of hub genes and connected TFs with R/Bioconductor packages.

## 3 Results

### 3.1 Characteristics of the datasets

In this study, 16 patients with stable ESRD (age: 47 ± 2 years) (mean ± SEM) and NHD (5–6 times a week, 6–8 hours per session) were enrolled. Detailed information on patients with ESRD, hematologic, and biochemical parameters before and after conversion to NHD were listed in the original article.^[6]^ We have used this microarray profile to obtain a global image of gene expression changes. Total RNA was isolated from peripheral whole blood before and after 3 months of established NHD. Gene expression before and after NHD was acquired through hybridization on Human HG-U133_PLUS2 GeneChip, and genes with DEGs were identified.

### 3.2 DEGs selection and hierarchical clustering analysis

**Figure 1:**
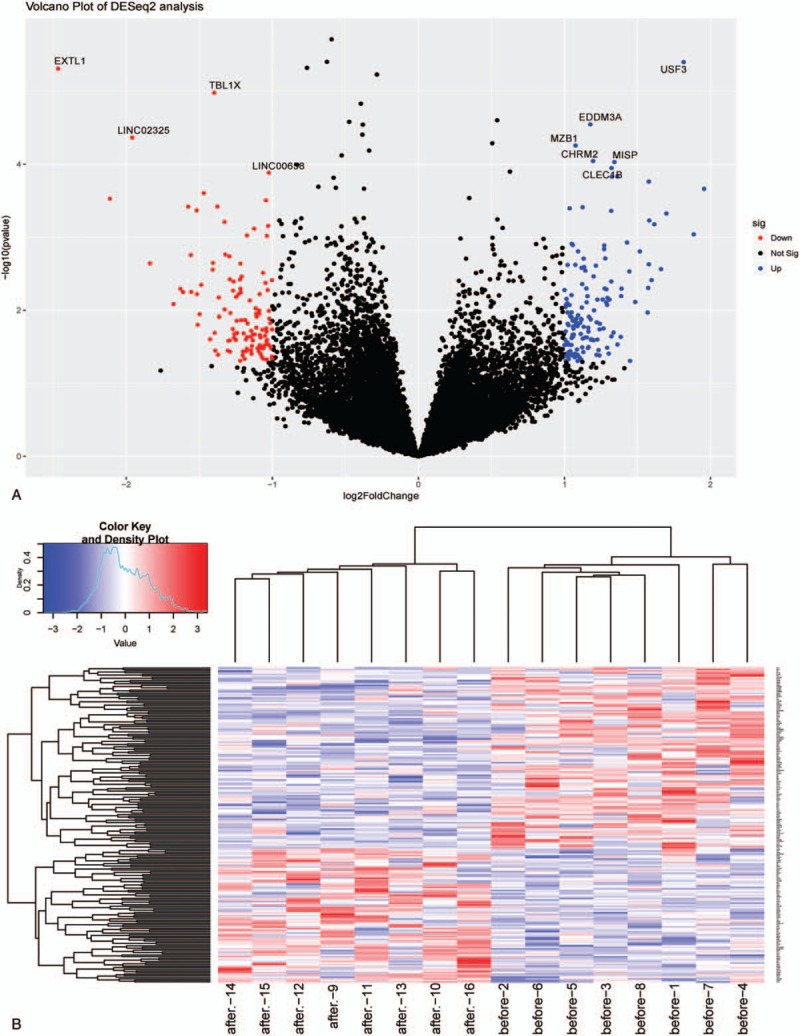
Summaries of different expression for mRNA sequence data. [(A) Volcano and (B) heatmap plots of different expressed miRNAs. In the volcano plots, each red color dot represents a downregulated or upregulated mRNA. In the heatmap plots, each row representing a probe and each column representing a sample. Expression levels are depicted according to the color scale, shown at the top. The red color indicated high expressed mRNAs and the blue color indicated low expressed mRNAs, above and below the median, respectively. The magnitude of deviation from the median is represented by the color saturation.]

Before calculating the DEGs, we removed the probes without corresponding annotation information. And then, we use the DESeq2 package for differential expression analysis, which was thought to be a robust method of analyzing RNA-Seq data.^[15,16]^ To determine the expression values of each gene, we used multiple GSE11227 probes corresponding to the median expression value of that gene. Finally, a total of 1486 genes were identified between the NHD group and the CHD group, including 570 upregulated genes and 916 downregulated genes (*P* < .05, Fig. 1A, Supplementary Table 1). Hierarchical clustering analysis of the 1486 DEGs was obtained from 16 samples of the patients with ESRD. The general gene expression patterns of the 2 groups were evidently different through heatmap view (Fig. 1B).

### 3.3 Gene co-expression networks construction

**Figure 2:**
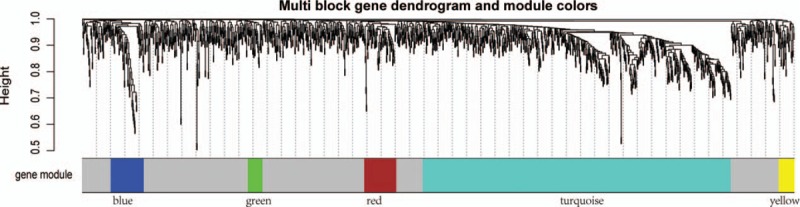
Summaries of weighted gene co-expression network analysis (WGCNA) for differentially expressed genes (DEGs). [Network analysis of gene expression in end-stage renal disease (ESRD) identifies distinct modules of co-expression genes. Each leaf (short vertical lines) in the dendrogram corresponds to a gene and the branches are expression modules of highly interconnected groups of genes with a color to indicate its module assignment. Modules are illustrated with different color obtained with different module detection sensitivity parameter called deepSplit.]

We selected the appropriate weighting parameter for the adjacency function, the soft-threshold β = 10, to construct gene modules using the WGCNA package. After determining the soft threshold, a total of 1486 DEGs were used to construct a WGCNA. According to the basic idea of WGCNA, we calculated the correlation matrices and adjacency matrices of gene expression profiles of NHD and CHD groups, and then transformed them into a TOM, and obtained the clustering dendrogram of DEGs. Together with TOM, we performed a hierarchical average linkage clustering method to determine the genetic module for each gene network (deep split = 2, cutting height = 0.99). In both the NHD and CHD groups, a total of 5 gene modules including 366 DEGs were recognized by dynamic tree cleavage (Fig. 2). Genes that not belong to any modules were placed in a gray module.

### 3.4 Biological process analysis of DEGs associated with immune functions

**Figure 3:**
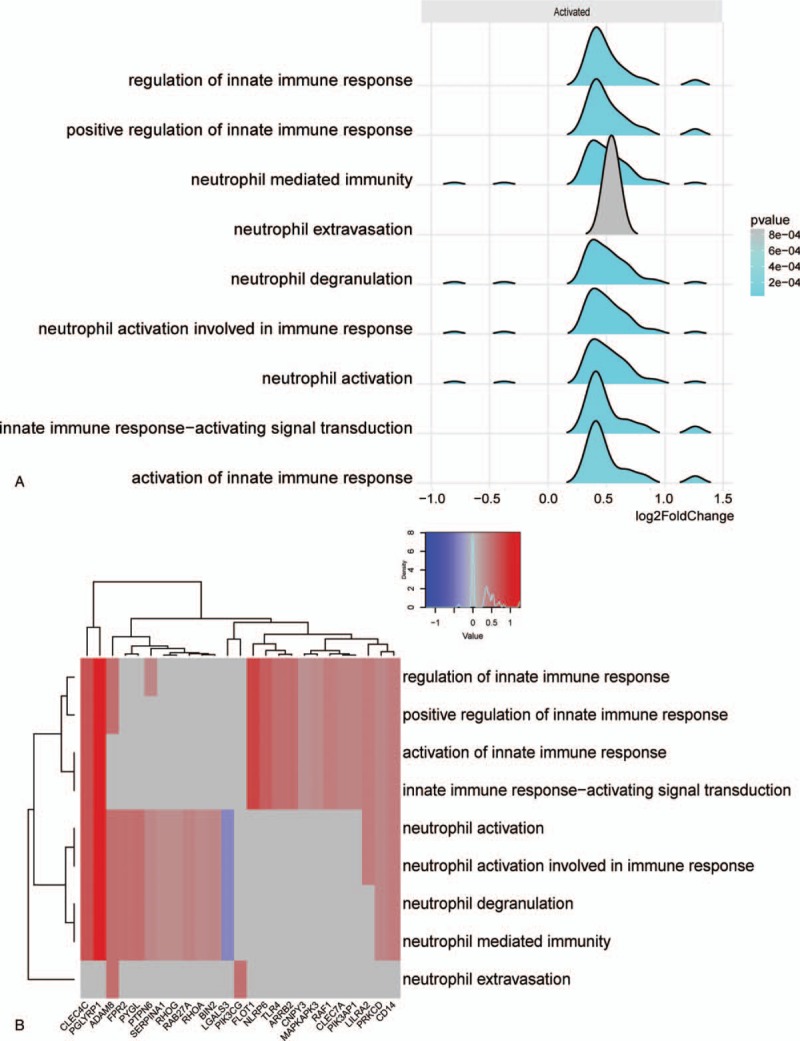
Overview of immunity-related biological processes of differentially expressed genes (DEGs). (A, Significant biological process of immune function. B, The enrichment map of immune biological process and core DEGs. The magnitude of gene counts compared all background genes is represented by the horizontal bar length. And the significant levels represented by the legend's color saturation.)

To reveal the biological processes and molecular function involved in NHD patients, we conducted a functional enrichment analysis. GO enrichment analysis of 366 DEGs was carried out by significant level *P* < .01. The most GO terminologies in biological processes were associated with neutrophil-mediated immunity, neutrophil degranulation, and neutrophil extravasation, as well as activation of innate immune response in the NHD group (Fig. 3A, Table 1).

**Table 1:**
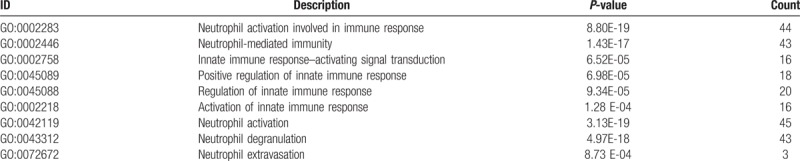
Immune function–related biological process.

In order to explore the intrinsic link between gene functions, we constructed a gene function regulatory network of the important GO terms (*P* < .01) in the biological processes. Then we found that hierarchical tree relationships between gene function was significant. The analysis showed that the gene function cascade eventually induced neutrophil degranulation (*P*_adj_ = 1.49e-12).

For a preliminarily test to assess whether the network was acceptable, we get the core corresponding genes of the 5 modules, and then performed heatmap for biological function associated immune activities. For the 5 modules, genes involved in neutrophil activation, extravasation and degranulation, and positive regulation of innate immune response were significantly clustered (Fig. 3B). It is suggested that this module is closely related to the occurrence and development of neutrophil degranulation and immune activation in ESRD, especially in patients with NHD. These results indicate that this module may be closely related to the immune response during ESRD.

### 3.5 Transcription regulatory network of hub genes

**Figure 4:**
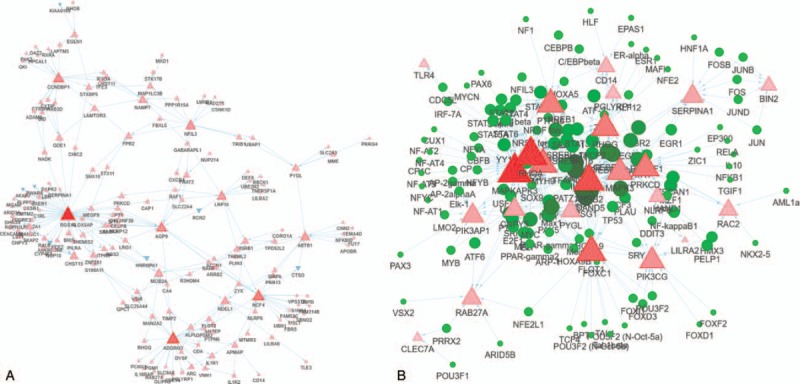
Transcription regulatory network of gene modules. [A, Hub genes in gene modules. B, The transcriptional regulators of main hub genes. The triangle represents the differentially expressed genes (DEGs) in each enriched module, the red and blue triangles represent the up- and downregulated DEGs, respectively. The size of the nodes was weighted by the power of the DEGs interacted with transcriptional regulators.]

These results suggest that the biological mechanisms of NHD patients are closely related to the neutrophil degranulation and immune activation. To screen the genes most relevant to immune function, we constructed a WGCNA of the 366 DEGs. Fourteen hub genes were found in the blue module response to immune function (Fig. 4A, Table 2) with a threshold of 0.2. Then, a transcriptional regulatory network of the 14 hub genes was constructed based on UCSC database, whose degree was >10. And we found a few core transcriptional regulators, including ARNT, C/EBPalpha, CEBPA, CREB1, PSG1, DAND5, SP1, GATA1, MYC, EGR2 and EGR3 (Fig. 4B, Table 2).

**Table 2:**
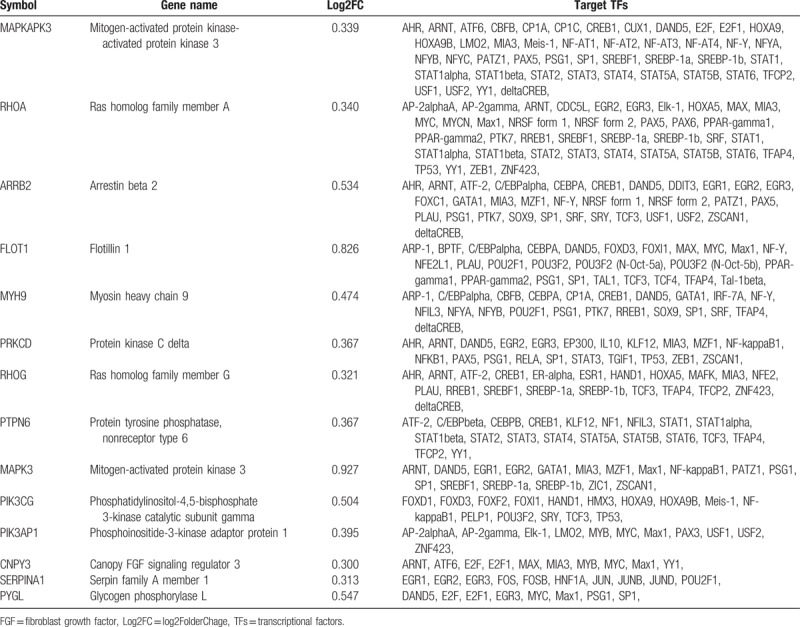
Overview of 14 hub genes and connected TFs.

## 4 Discussion

The main purpose for this study was to use a global approach to construct a gene co-expression network that predicts candidate gene clusters involved in the immune functions of ESRD. Although appropriate hemodialysis treatment of ESRD has successfully improved quality of life and reduced the incidence and mortality of ESRD to some extent,^[17]^ there is still a need for effective methods to prevent adverseness and prolong patients’ life.

WGCNA is a powerful approach to identify gene modules as candidate biomarkers or therapeutic targets based on co-expression network.^[18]^ Comparing any other analytical methods, WGCNA facilitate the summary and standardization of methods and functions, including weighted and unweighted correlation networks, and is successfully used to identify pathway-related gene modules and hub genes of NHD in ESRD. In WGCNA, we identified 5 gene modules based on 1486 DEGs. By functional enrichment analysis, the modules have obvious biological significance, which were significantly enriched in immune activation, neutrophil degranulation, and extravasation.

Through the enrichment function analysis of the gene modules, we determined that accompanied by the activation of granulocyte signal pathway and the initiation of immune system on NHD in patients with ESRD, so that platelet activation and blood coagulation. In addition, patients on NHD have normal plasma phosphate levels without the restrictions in dietary.^[19]^ These findings have a potentially positive impact on our known about the complexity and significant immune mechanism of ESRD.

A dialyzer and dialysate are required for hemodialysis, which is susceptible to contamination by aquatic bacteria such as gram-negative bacilli, and requires adequate attention to prevent endotoxin contamination such as microorganisms. In addition to that, infection is the second leading cause of death in hemodialysis patients, and infection tends to be severe in these patients.^[20]^ Clinical data shown that the prevalence of tuberculosis is high among hemodialysis patients and that infections such as flu syndrome among hemodialysis patients are difficult to cure and tend to persist and follow a severe course, and that the incidence of malignant tumors is higher among hemodialysis patients than among individuals with intact renal function.^[21]^ Our results suggest that NHD has altered several genes expression in response to immunity by neutrophil activation, degranulation, and extravasation. TLR-2 and TLR-4 have been reported to be associated with the compromised immune function in NHD patients.^[22]^

Neutrophils account for approximately 50% to 70% of all white blood cells and are the most abundant white blood cells in humans. Studies have found that neutrophils tend to engulf carbohydrates in bacteria, and increase the phagocytic capacity to engulf bacteria when simple sugars are digested.^[23]^ Neutrophils are highly motorized and are attracted by cytokines expressed by activated endothelium, mast cells and macrophages, and can rapidly accumulate in infected lesions. Neutrophils express and release cytokines, which in turn enhance the inflammatory response in several other cell types.^[24]^ In addition to recruit and activate other cells of the immune system, neutrophils play an important role against invading pathogens in the first-line defense. Neutrophils have 3 methods for directly attacking the invasion of pathogens: phagocytosis, degranulation, and generation of neutrophil extracellular traps.^[25]^ And our analysis results suggest that neutrophils, whose activation, degranulation, and extravasation have an important positive regulatory effect on immune responses.

Although hemodialysis has been developed almost 1 century ago, early use was complicated by the clotting of dialysis circuit.^[26]^ Hemodialysis becomes feasible for a large population until heparin is introduced into anticoagulation circuit.^[27]^ Hemodialysis patients have an increased tendency to bleed due to the accumulation of uremic toxins that cause platelet dysfunction, especially anticoagulation with heparin.^[28,29]^ Paradoxically, in our analyzed results, the risk of blood coagulation and platelet activation and aggregation increased in NHD patients. Perhaps this paradox phenomenon stems from the disturbance of endothelial damage and the metabolism, expression, and activity of certain procoagulant factors.^[30]^

Additional analytic results of genetic alterations revealed a few core markers may play an important role in the biological process of immune response in patients with ESRD. For example, cAMP response element-binding protein (CREB) can bind to certain DNA sequences to increase or decrease the transcription of genes containing cAMP-responsive elements.^[31]^ In addition, CREB can promote anti-inflammatory immune responses by inhibiting NF-kB activity and producing T-regs, thereby inhibiting inflammation, tissue damage, and autoimmune responses, or pathogenic infection factors.^[32]^

In summary, this study used transcriptional network analysis to identify co-expression modules for the first time. Significant gene modules and biological process were revealed to make a powerful comprehensive about the complexity mechanism in patients with ESRD. The limitation of this study is that the sample size is relatively small and there is no experimental validation of the core genes and TFs, therefore, further extensive experiments need to be performed to confirm the results of this study.

## Author contributions

**Data curation:** Hongwei Dai.

**Formal analysis:** Hongwei Dai.

**Investigation:** Hongwei Dai.

**Methodology:** Jiao Zhou.

**Software:** Jiao Zhou.

**Supervision:** Bo Zhu.

**Validation:** Jiao Zhou.

**Visualization:** Jiao Zhou.

**Writing - original draft:** Hongwei Dai, Bo Zhu.

**Writing - review and editing:** Bo Zhu.

## Supplementary Material

Supplemental Digital Content
